# The Relationship between Spatial Patterns of Illnesses and Unemployment in Iraq-2007

**DOI:** 10.5539/gjhs.v4n1p192

**Published:** 2012-01-01

**Authors:** Faisal G. Khamis

**Affiliations:** AL-Zaytoonah University of Jordan / Faculty of Economics and Administrative Sciences E-mail: faisal_alshamari@yahoo.com

**Keywords:** Spatial autocorrelation, Sharing boundary neighbors, Mapping, Global and local moran, Monte carlo simulation, Unemployment, Chronic illnesses

## Abstract

Studies of the relationship between spatial patterns of chronic illnesses (CI) and unemployment rate (UR) characteristics were not well documented. However, when analyzing the data that were collected on geographic areas, the spatial effects were seldom considered. This study addresses this concern by applying the mapping and spatial analysis techniques in studying how UR pattern is related to the CI pattern in Iraq. The aim is to assess the existence of spatial pattern in CI across geographical areas, and find whether this pattern was influenced by the pattern of socioeconomic indicators such as UR. The study design was cross-sectional census data obtained in 2007. Governorates were used as the respective units of the analysis. Two statistics of spatial autocorrelation based on sharing boundary neighbours known as global and local Moran measures were used to investigate the global and local clustering respectively. To investigate the bivariate spatial relationship between CI and UR, Wartenberg’s (1985) measure was used. It was found that UR varied significantly across different governorates, while CI didn’t. Significant local clusters in UR, in northern and southern parts of the country were found, while no significant local clusters were found in CI. No significant spatial association was found between CI and UR based on bivariate spatial correlation coefficient.

## 1. Introduction

Unemployment is one of the main socioeconomic issues that negatively affect both economic activity and social life. In recent years, there has been a growing interest in examining the existence of spatial autocorrelation of UR and its spatial relationship with many indicators such as poverty, education, health, etc. Low wage flexibility and limited labour mobility involve persistent unemployment differentials across governorates in Iraq. The present paper focuses on the spatial structure of UR disparities across governorates and its relationship to CI disparities.

Regions, independent of their geographic level of aggregation, are known to be interrelated partly due to their relative locations. Governorates are tightly linked by migration, commuting, and enter-governorate trade. These types of spatial interactions are exposed to the frictional effects of distance, which could cause the spatial dependence of governorate labour market conditions. Similar economic performance among governorates can be attributed to proximity. Benach *et al*. (2009) stated that the employment conditions and associatedwork organization of most migrant workers are dangerous to their health inequalities. For example, theimpact of Korean employment conditions on workers’mental health is worse for immigrants, minorities and unionmembers because of their labor market vulnerability and thehostility of management to union organizers and members. The study of Wahl *et al*. (2009) in Norway supported the assumption of a complex and indirect relationship between chronic pain and global quality of life (GQOL). Wahl *et al*. found negative effect of CI on GQOL. The CI is a major cause of disability (Wehdell, 2001). Smith (2008) in a least-squared regression model found that UR has a moderate effect on infant mortality rates.

The UR was studied in many countries using different statistical measures. Given the large literature documenting the important relationship between employment outcomes and health, Johnston and Lordan(2011) concluded that this is a potentially important pathway through which discrimination affects health. Evidence provided by Eichengreen (1993) indicated that the responsiveness to health, social, and other factors of unemployment differentials was much greater in US than in Europe. Bertola (1999), as well as Balu and Kahn (1999), analyzed the impact of different institutions and regulations on labour market outcomes. According to their results, wage adjustment and labour mobility wereaffected by minimum-wage provisions unemployment benefits and welfare payments. However, the results of Layard (1997) implied that strict labour market regulations, employment protection and minimum wages were not the main target areas of policies aiming at a significant decline of unemployment. Instead they advised reform of social security systems combined with active labour market policies.

In US, unemployment rates remained near a 25-year high and global unemployment was rising (Roelfs *et al.*, 2011). In Europe and Chile, Jeffers, *et al*. (2010) found that unemployed individuals have poorer mental health than the employed and possible subsequent major depression, which could lead to suicide. Being unemployed were considereda major risk factor causing suicide by several authors (Chida, 2006; and Hawton & Herringen, 2009). Jin *et al.*, (1995) found a strong positive association between unemployment and health problems, such as morbidity at the population level. Tontsa *et al*. (2011) concluded that socioeconomic wellbeing across 33 small mining towns in Western Australia is highly variable. Eriksson *et al*. (2010) addressed unemployment and related social factorsas risk factors for impaired mental illness in Denmark. They found that experiences of long- and medium-termunemployment are followed by an increased probability of the individualbeing admitted for the first time to a psychiatric hospital.

The relationship between unemploymentand health is expected in the poorest areas of the world, and morespecifically in countries with severe economic and socialcrises, such as war, poverty and mass migrations in Iraq, Afghanistan, and Africa South of the Sahara (Benach *et al*. 2009). In Iraq unemployment remained at very high levels. The most affected are women and young people (Agency for Technical Cooperation and Development, 2010). Epilepsy is a chronic illness that affects all ages andhas long-term complications, includingun-employability (AL-Saad, *et al*. 2001). Their study was conducted in Salahuddin governorate. Unemployment in this governorate was found 33%, and this rate was significantly greater among those stricken by epilepsy especially the young. Youth unemployment is high and increasing: 57% of those aged between 15 and 29 are unemployed and 450000 young men are new entrants of labour market each year (UNIAU Iraq labour force analysis 2003-2008).

A common feature of most of the above mentioned studies is that they investigated the functioning of labour market adjustments and the effects of labour market regulations without considering the spatial dimension of area labour market disparities. Most of these studies have shown that unemployed persons have an increased risk of death. Some studies investigated the wage curve taking spatial effects into account. Manning (1994) and Buettner (1999) analyzed the relationship between earnings and unemployment for British countries and German regions respectively. An analysis by Molho (1995) confirmed that there is significant spatial interaction among regional labour markets in UK. Overman and Puga (2002) analyzed unemployment clusters across European regions. The results of their nonparametric approach indicted that unemployment rates were much more homogeneous across neighbouring areas than across regions in the same EU country. The results found by Filiztekin (2007) indicated that the provincial unemployment rates were quite persistent and the gap across different regions widens further with spatial clusters emerging across Turkey. Lopez-Bazo, Barrio and Artis, (2000) discussed the role of neighbouring effects in explaining the spatial distribution of unemployment, where their results pointed to the emergence of at least two clusters in the regional distribution of UR in Spain.

To understand the linkages between socioeconomic variables, investigations should focus on features of the areas rather than on the compositional characteristics of residents of the area, which cannot fully describe the social environment in which people live (Macintyre, Maciver & Sooman, 1993). So, spatial autocorrelation and geographical pattern of UR and CI were studied using lattice data. Spatial autocorrelation is the term used for the interdependence of the values of a variable over space. However, it was argued that lattice data are spatially correlated, where exploratory spatial data analysis (ESDA) was used using lattice data. The ESDA quantifies the spatial pattern in order to increase the analyst’s knowledge of the spatial system. As well as mapping plays an important role in the monitoring of unemployed people and CI. Maps can reveal spatial patterns not previously recognized or suspected from the examination of a table of statistics and reveal high risk communities or problem areas (Lawson & Williams, 2001). However, the purpose of spatial analysis is to identify pattern in geographic data and attempt to explain this pattern. Findings are expected to enhance unemployment and CI monitoring and policing interventions across governorates in Iraq.

Available evidence was shown that employment conditions and associated work organization of most migrant workers are dangerous to their health (Benach *et al*. 2009). Reducing UR and CI inequalities are not a primary objective but emergent prosperity is. The importance of this objective emanates the argument that unemployment is a standard indicator of poverty which means that poor people cannot pay for medical treatment especially those who suffered from CI. Also the job prospects for the people who suffered from CI may be limited. Cattaneo, (2006) stated that a strong link existed between poverty and unemployment, being the lack of employment one of the main determinants of poverty. According to the study in Jordan by Amerah, (1993), health was affected negatively by unemployment. Elhorst, (2003) proposed several reasons that make studying the spatially uneven distribution of unemployment worthwhile. One of these reasons is that unemployment differentials imply inefficiency in the economy as a whole and reduce growth. To the author’s knowledge no studies used the spatial analysis techniques and geographical mapping in studying the inequalities in UR and CI gradient and their relationship to each other in Iraq. Furthermore, the studies that used advanced statistical techniques, such as structural equations modeling, in examining the inequalities of UR and CI were very limited in Iraq.

The importance of mapping was stated by Koch (2005): why make the map if detailed statistical tables carry the same results? Perhaps the most important reason for studying spatial statistics is not only interested in answering the “how much” question, but the “how much is where” question (Schabenberger&Gotway, 2005). In light of these: (1) the existence of spatial global clustering and (2) spatial local clusters of governorates with respect to UR and CI were investigated. Also, the bivariate spatial association between UR and CI was examined. Thisstudy contributes to the literature by examining the geographical distribution of UR and CI, spatial global clustering, and local clusters of UR and CI. The study design was a cross-section analysis in a census survey conducted in Iraq in 2007.

The data analysis follows five steps. In step 1, the levels of UR and CI were visually inspected using the quartiles based on choropleth mapping. Step 2 includes the calculation of global Moran’s *I* for each UR and CI to detect the global clustering and also the significance of *I* statistic was examined using permutation test. Step 3 involves the calculation of local Moran’s *I_i_* for each ithgovernorate to detect the local clusters of UR and CI, and also the *p*-values of local Moran’s *I_i_* values were calculated using Monte Carlo simulation. In step 4 the gradients of quartiles of local Moran values were visually inspected. In Step 5, the bivariate spatial correlation between UR and CI was examined based on Wartenberg’s (1985) statistic. In conclusion, spatial global clustering was found for UR but was not found for CI. Local clusters in UR were found but for CI were not found. The major contribution was the demonstration that spatial locations had statistically significant effects on the likelihood and disparity of UR.

## 2. Materials and Methods

**Data:** Data were collected from Iraq Household Socioeconomic Survey, based on a census conducted in Iraq in 2007. For (*N* = 18) governorates, transformed UR and CI variables were used. The UR is defined as the percentage of unemployed persons in the total economically active population (the total of unemployed and employed persons). An unemployed person is a person aged (15-65) years who are without work, able to work, available for work, actively looking for work, and willing to accept the market wage. The percentages ofpersons reported to have CI in each governoratewereapplied. The CI included the following: Diabetes, hypertension, heart disease, kidney, Tumors and high cholesterol, psychiatric and nervous sensory,stomach, intestines, ulcers, Thyroid, hepatitis, respiratory and chest, gynecological, hematological, Skin, venereal, parasitic and other, Urinary and genital, and Impotency and other.

**Analysis:** Data analysis involved five steps. In step 1, the UR and CI were tested for normal distribution. They weren’t found to follow normal distribution. Therefore, both variables were transformed to follow normal distribution using LISREL software. The LISREL scales the normal scores so that the transformed variables have the same sample mean and standard deviation as the original variable. Thus, the normal score is a monotonic transformation of the original score with same mean and standard deviation (this characteristic can be considered as an advantage in this transformation) but with the values of skewness and kurtosis much reduced. In step 2, visual inspection based on the quantified gradients for transformed UR and CI using quartiles were conducted. Step 3 included the calculation of global Moran’s *I* for transformed UR and CI to detect the global clustering and also the significance of *I*-statistic using permutation test for each variable was examined. Step 4 involved the calculation of local Moran’s *I_i_* for the *ith* governorate and it’s *p*-value using Monte Carlo simulation to detect the local clusters fortransformed UR and CI. In step 5, using quartiles, visual inspection of local Moran values for each variable was inspected based on choropleth mapping.

**Figure 1:**
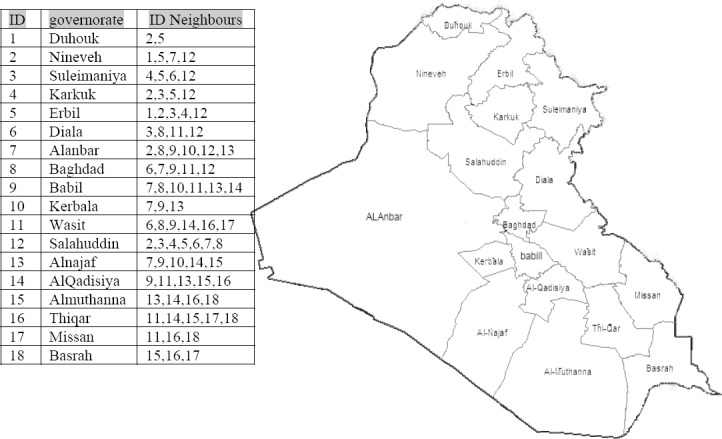
Study area shows all governorates with their ID and the neighbours of each governorate

The UR and CI values were categorized into four intervals. These intervals were used for all maps using darker shades of gray to indicate increasing values of UR and CI. Such approach enables qualitative evaluation of spatial pattern. In the neighbourhood researches, neighbours may be defined as governorates which border each other or within a certain distance of each other. In this research neighbouring structure was defined as governorates which share a boundary. The *second order* method (queen pattern) which included both the first-order neighbours (rook pattern) and those diagonally linked (bishop pattern) was used. A neighbourhood system was given in Fig. 1, where ID neighbour for each governorate was shown. Although maps allow visual assessment of spatial pattern, they have two important limitations: their interpretation varies from person to person, and there is the possibility that a perceived pattern is actually the result of chance, and thus not meaningful. For these reasons, it makes sense to compute a numerical measure of spatial pattern, which can be accomplished using spatial autocorrelation. Therefore, global spatial clustering and local spatial clusters were identified.

*Identification of global spatial clustering:* The goal of a global index of spatial autocorrelation is to summarize the degree to which similar observations tend to occur near to each other in geographic space. In this exploratory spatial analysis, the spatial autocorrelation using standard normal deviate (z-value) of Moran’s *I* under normal assumption was tested. Moran’s *I* is a coefficient used to measure the strength of spatial autocorrelation in regional data. Global clustering test was used to determine whether clustering has existed throughout the study area, without determining statistical significance of local clusters. Moran’s *I* is calculated as follows (Cliff and Ord, 1981):


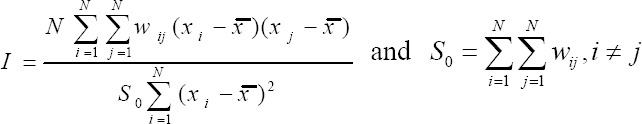


where, *N* = 18 is the number of governorates, *w_ij_* = 1 is a weight denoting the strength of the connection between two governorates *i* and *j* that shared a boundary, otherwise, *w_ij_* = *zero*, *x_i_* and *x_j_* represent the transformed UR or CI in *ith* and *jth* governorate respectively. The autocorrelation coefficient can be used to test the null hypothesis of no spatial autocorrelation or spatially independent versus the alternative of positive spatial autocorrelation:


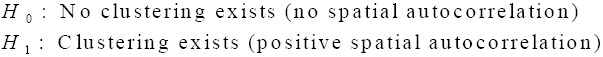


A significant positive value of Moran’s *I* indicates positive spatial autocorrelation, showing the overall pattern for the governorates having a high/low level of UR or CI similar to their neighbouring governorates. A significant negative value for Moran’s *I* indicates negative spatial autocorrelation, showing the governorates having a high/low level of UR or CI unlike neighbouring governorates. To test the significance of global Moran’s *I , z*-statistic which follows a standard normal distribution was applied. It was calculated as follows (Weeks, 1992):


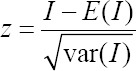


Permutation test wasapplied. A permutation test tells us that a certain pattern in data was or was not likely to have arisen by chance. The observations of each UR and CI were randomly reallocated 1000 times with 1000 of spatial autocorrelations were calculated in each time to test the null hypothesis of randomness. The hypothesis under investigation suggests that there will be a tendency for a certain type of spatial pattern to appear in data, whereas the null hypothesis says that if this pattern was present then this was a pure chance effect of observations in a random order. The analysis suggested an evidence of clustering if the result of the global test was significant but it didn’t identify the locations of any particular clusters. Beside, clustering which represent global characteristic, the existence and location of localized spatial clusters in the study population are of interest in geographic sociology. Accordingly, local spatial statistic was advocated for identifying and assessing potential hot spots or clusters.

*Identification of local spatial clusters:* A global index can suggest *clustering* but cannot identify individual *clusters* (Waller & Gotway, 2004). Anselin, (1995) proposed the local Moran’s *I_i_* statistic to test the local autocorrelation, where local spatial clusters, sometimes referred to as hot spots, may be identified as those locations or sets of contiguous locations for which the local Moran’s *I_i_* was significant. Anselin stated that the indication of local patterns of spatial association may be in line with a global indication, although this is not necessarily the case. It is quite possible that the local pattern is an aberration that the global indicator would not pick up, or it may be that a few local patterns run in the opposite direction. However, Moran’s *I_i_* for *ith* governorate may be defined as (Waller & Gotway, 2004):


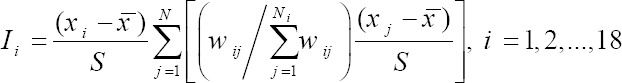


where, analogous to the global Moran’s *I*, the *x_i_* and *x_j_* represents the UR or CI in *ith* and *jth* governorate respectively, *N_i_* = number of neighbours for *ith* governorate, and *S* is the standard deviation. It was noteworthy that the numbers of neighbours for *ith* governorate were taken into account in *I_i_* statistic by the amount:


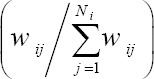


where *w_ij_* was measured in the same manner as in Moran’s *I* statistic. Local Moran statistic was used to test the null hypothesis of *no clusters*. However, local Moran statistic is a decomposition of global Moran’s *I* into the contributions of small areas.

Clusters may be due to aggregations of high values, aggregations of low values, or aggregations of moderate values. Thereby, high values of *I_i_* suggested clusters of similar (but not necessarily large) values across several governorates, and low value of *I_i_* suggested an outlying cluster in a single governorate *i* (being different from most or all of its neighbours). A positive local Moran value indicates local stability, such as governorate that has high/low UR surrounded by governorate that has high/low UR. A negative local Moran value indicates local instability, such as governorate has low UR surrounded by governorate has high UR or vice versa. However, each governorate’s *I_i_* value can be mapped to provide insight into the location of governorates with comparatively high or low local association with its neighbouring values. In the statistical analysis, all programs performed in S+8 Software.

*Bivariate spatial association:* So far, spatial method have only presented that quantified the spatial structure of one variable at a time. There is controversy about the appropriate measure for bivariate spatial association. However, spatial dependence or spatial clustering causes loss in the information that each observation carries. When *N* observations were made on a variable that was spatially dependent (and that dependence was positive so that nearby values tend to be similar), the amount of information carried by the sample was less than the amount of information that would be carried if the *N* observations were independent, because a certain amount of the information carried by each observation was duplicated by other observations in the cluster. A general consequence of this was that the sampling variance of statistics was underestimated. As the level of spatial dependence increases the underestimation increases. The problem is when spatial autocorrelation is present, the variance of the sampling distribution of e.g., Pearson correlation coefficient which is a function of the number of pairs of observations, is underestimated. Spatial autocorrelation coefficients are therefore modified to estimate the spatial correlation between two variables (Wartenberg 1985):


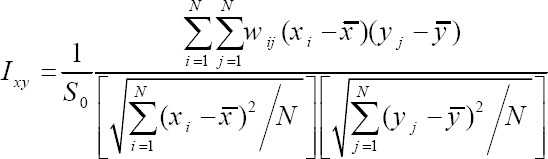


Where *x* and *y* are the UR and CIvariables respectively. Although the mathematics is quite straightforward, very few software packages allow computing *I_xy_* (Fortin & Dale, 2005). Thus, programming was used to find the value of *I_xy_*. To test the significance of *I_xy_*, *z*-statistic was applied which follows approximately standard normal distribution:


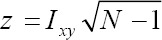


## 3. Results

Descriptive analyses were performed to assess the demographic characteristics of the data set. The mean and standard deviation for transformed URwere found 11.71 and 4.83 respectively; skewness and kurtosis were found 0.00 and -0.10 respectively. The five-number summary of transformed UR data set consists of the minimum, maximum and quartiles written in increasing order: Min=2.14, Q_1_=8.29, Q_2_=11.71, Q_3_=15.14 and Max=21.28. From the five-number summary, the variations of the four quarters of UR data were found 6.15, 9.57, 3.43 and 6.68 respectively, where the second quarter has the greatest variation of all. Also, descriptive statistics were calculated for transformed CI, where the mean and standard deviation were found 9.94 and 7.11 respectively; skewness and kurtosis were found 0.00 and -0.10 respectively. The five-number summary of transformed CI data set was found: Min=-4.13, Q_1_=4.91, Q_2_=9.70, Q_3_=14.08 and Max=24.02. From the five-number summary, the variations of the four quarters of CI data were found 9.04, 4.79, 4.38 and 9.94 respectively, where the fourth quarter has the greatest variation of all.

Thiqar governorate accounted for the highest UR (20.9%). It was followed by the governorates Diala and Missanwhich accounted for (20.4%) and (19.6%), respectively. The lowest rate was in the governorate of Suleimaniya with (2.14%). This can be explained by the persistent growth of economic activity in most fields, which provide more job opportunities. However, the UR in Iraq decreased dramatically from 28.10% in 2003 to 11.71% in 2007. Suleimaniya governorate accounted for the highest CI (29.6%). It was followed by the governorates of ALnajaf and ALqadisiyawhich accounted for (21.5%) and (16.5%), respectively. The lowest CI was in the governorate of Karkuk with (2.2%).Figure 1 shows the study area explaining all governorates with their identification numbers (ID).

**Figure 2:**
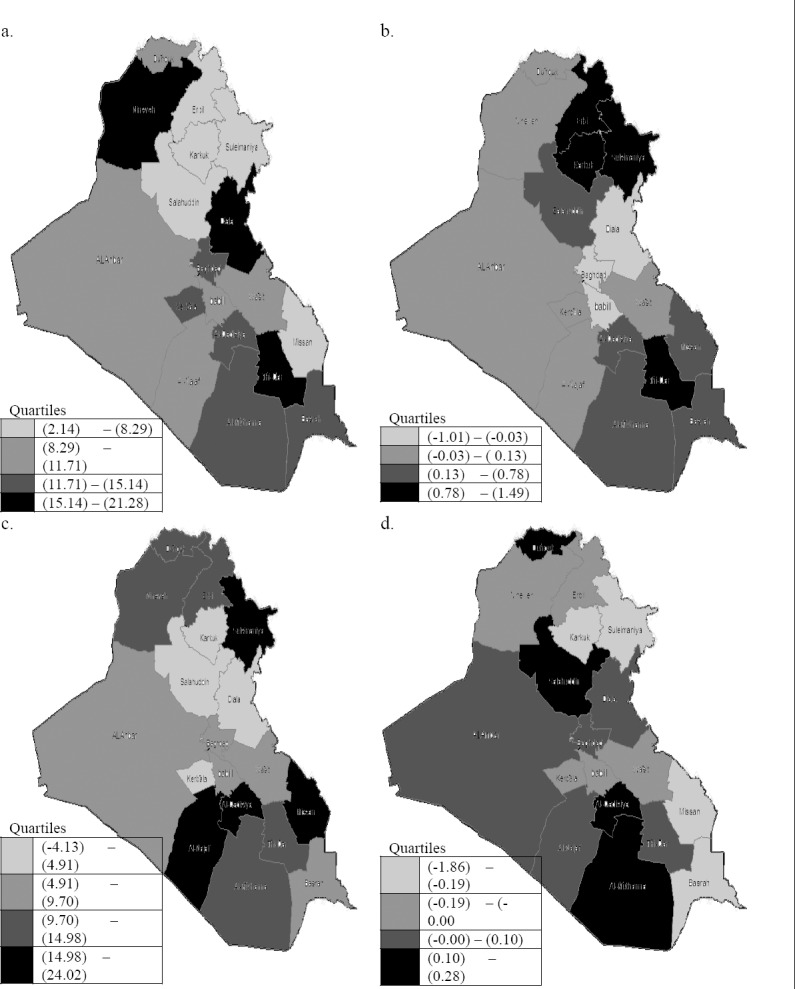
Choropleth maps show: a. transformed UR variable, b. local Moran values of UR variable, c. transformed CI variable, and d. local Moran values of CI variable

Since the local Moran’s *I_i_* varied by location, it was easier to interpret visually by color coding of each governorate. Maps displayed geographical inequalities across governorates of Iraq. Figures 2a, b, c, and d show visual insight for transformed UR, and its local Moran values, transformed CI and its local Moran values respectively, with the darkest shade corresponding to the highest quartile. Based on visual inspection, an overall worsening pattern (higher scores) for UR was found in the western-northern, mid, and southern parts of the country. The suggestion of spatial clustering of similar values for UR was confirmed by a positive significant global Moran’s *I* of 0.23 with an associated standard normal *z*-value of 2.25 and *p*–value = 0.024. Also, based on visual inspection, an overall worsening pattern (higher scores) for CI was found in the northern and southern parts of the country. The suggestion of spatial clustering of similar values for CI was not confirmed by a negative global Moran’s *I* of -0.12 with an associated standard normal *z*-value of -0.48 and *p*–value = 0.632. The high level of CI in somegovernorates such as 14 and 15 could probably be contributed by the high level of UR in these governorates, in some of their neighbours,and/or by the UR inequality among their neighbours as shown in Fig. 2a and 2c. Seven significant clusters have high level in UR were found as shown from their *p*-values, where their ID (3, 4, 5, 12, 15, 16, and 17). For CI, no significant clusters were found.

To investigate global clustering, permutation test was done, where the permutation *p*–value=0.018for transformed UR was found significant; while *p*–value=0.636for transformed CI was found not significant. Thus, the null hypothesis of no spatial autocorrelation was rejected for UR but not rejected for CI. The results of local Moran’s *I_i_* values for transformed UR and CI, and their p-values are reported in Table 1.

**Table 1:** Shows both transformed UR (%) and CI (%), Local Moran’s *I_i_* values for UR and CI, and their corresponding *p*-values

ID	Transformed UR	*I_i_* for UR	*p*-value	Transformed CI	*I_i_* for CI	*p*-value
1	10.02	0.09	.344	11.41	0.10	.333
2	15.80	-0.65	.925	12.43	-0.07	.615
3	2.14	0.95	**.011**	24.02	-1.86	.999
4	6.55	1.49	**.004**	-4.13	-0.84	.940
5	5.12	0.93	**.007**	14.66	-0.11	.666
6	18.31	-1.01	.975	2.35	0.01	.431
7	9.30	0.03	.342	7.46	0.04	.312
8	12.04	-0.01	.493	8.97	0.09	.260
9	8.51	-0.01	.480	8.97	-0.01	.500
10	13.05	-0.08	.613	3.92	-0.16	.703
11	10.71	-0.15	.717	6.39	-0.05	.587
12	7.62	0.32	**.058**	0.24	0.11	.201
13	11.38	0.00	.462	19.65	0.06	.301
14	14.13	0.17	.181	17.54	0.28	.112
15	14.91	0.45	**.076**	13.50	0.23	.167
16	21.28	0.91	**.006**	10.43	0.02	.396
17	16.87	0.73	**.046**	15.97	-0.31	.788
18	13.05	0.34	.135	5.23	-0.31	.785

The Pearson correlation coefficient between UR and CI variables was found negative (-.06), which is not significant with (*p* = 0.809). Bivariate spatial correlation between the UR and CI was found (*I_xy_* = –0.01) which is not significant with (z=-0.04 and *p* = 0.569). However, although both results were not significant, Pearson coefficient is always over estimated when used in finding the spatial correlation. That’s why, in investigating the bivariate spatial correlation, it is recommended to use Waternberg (1985) measure.

## 4. Discussion

The association between the spatial patterns of UR and CI was examined, allowing for the effects of neighbouring governorates that share the boundary with a particular governorate. Findings allow policy makers to better identify what types of resources are needed and precisely where they should be employed. The above framework is proposed to analyze the spatial pattern of CIand revealed some noteworthy findings. The rationale behind the relationship between CI and UR is that unemployed people usually suffer from financial strain that could be caused by increased health problems.

After rejecting the null hypothesis, it becomes possible to conclude that there is some form of clustering. It is of course of interest to know the exact nature of the clustering process. Is it only global type clustering or are there hot-spot clusters? If the later, how many hot-spots are there and where are they located? Our analysis of the association between CI and UR used exploratory tools such as descriptive tables and small area choropleth maps. Geographical distributions of CI and UR in quantiles were examined visually using maps.

Research on neighbourhoods and health is motivated by the idea that we live in places that represent more than physical locations. They are also the manifestation of the social, cultural, political and geographic cleavages that shape a constellation of risks and resources. The first wave of studies on neighbourhoods and health focused on showing the relevance ofneighbourhoods and the effects beyond individual socioeconomic characteristics. These studies argued that neighbourhoods influence health by behavioralpatterns such as collective socialization, peer-group influence, and institutional capacity. The second wave of the studies evaluated these mechanisms with latent measures of neighbourhood characteristics, such as level of segregation, collective social and economic capacity (Sampson *et al*. 2002).

The URmay be associated with CI reflectingtheexisting of individual income which provides good medical care, high quality of food, and acceptable household conditions. The usual correlation coefficients, such as Pearson, only test whether there is an association between two attributes by comparing values at the same location. Map comparison involves more than pair wise comparisons between data recorded at the same locations as spatial units were arbitrary subdivisions of the study region and people could move around from one area to another and could be affected by UR levels in areas other than the area they live in. i.e., the level of CI in *ith* governorate was thought to be influenced by the levels of UR not just in *ith* governorate but also in neighbouring governorates. Neighbourhood residential turnover had been linked to poor child development, problem behavior, and health risks (Jelleymanand Spencer, 2008).

Permutation distribution can be used to test the significance of the global Moran statistic, for this purpose 1000 random permutations were used. The *p* = 0.018 for Morans’s *I* of UR and *p* = 0.636 for Morans’s *I* of CI were found. Simulated data is useful for validating the results for such analysis. However, using Monte Carlo simulation, 9999 random samples were simulated, 18 values for each sample, for both CI and UR. These samples (9999 matrices, each had two columns) were generated under bivariate normal distribution.

As stated by Charloit *et al*. (2005), CI is one of the indicators that measure children’s health in Denmark and Sweden. They found that children in families with one or both parents without paid work had an increased prevalence of recurrent psychosomatic symptoms and CI. The essay of Bambra (2011) showed that the relationship between work, worklessness, and health inequalities was influenced by a broader political and economic context in the form of welfare state regimes. Public health initiatives could target unemployed persons for more aggressive cardiovascular screening and interventions aimed at reducing risk-taking behaviors (Roelfs, *et al.*, 2011). When people are unemployed they have less money to spend on medical treatment and paydoctors’ bills, purchase healthy food, water, and shelter. All of which leads to a higher risk of CI. Better allocation of resources to healthcare and health awareness can help lead to lower levels of CI. One cannot believe that these problems will ever be fully resolved. However, with more effort, time, and better allocation of resources, an enormous impact can be achieved.

When UR is low, unemployed individuals may have special problems or characteristics that make them more vulnerable to ill health (Martikainen and Valkonen, 1996). The analysis showed that several governorates (4, 6, 8, and 10), confirmedthe findingsof Martikainen and Valkonen. However, one could also argue that being unemployed in times of low levels of unemployment may lead to a social stigma to a greater extent than when unemployment levels are high, which may partly explain the increased risk of ill health.

Although this study could not confirm any causation, there are two possibilities. First, UR could be caused CI or second, vice versa. Epidemiologic evidence suggests that the direction of causation from unemployment to illness has a greater possibility than the converse (illness causes unemployment). Although more research can be done to elucidate mechanisms and mediating factors, the present author found sufficient evidence to recommend that intervention research, to determine ways to reduce the adverse effect of unemployment on health, be a priority. Unemployment may exert detrimental effects on health through many mechanisms: (1) by disrupting community and personal social relationships (Jackson, 1993), (2) by leading to greater risk behavior, such as alcohol consumption and poor diet (Morrell *et al*. 1993), (3) by causing stress (Moser, 1986), and (4) by precipitating reaction, like that caused by other losses (Jackson, 1993). It didn’t assess the evidence for any particular mechanism or series of mechanisms since the main purpose was to assess whether, not how, UR pattern is related to the pattern of CI.

## 5. Conclusion

Clustering of transformed UR and CI are studied, and the spatial association between them. The association was not found significant based on Wartenbergcoefficient. Although, a causal relationship between UR and CI cannot be provided, the results are conclusive in at least five aspects: First, based on mapping the quartiles of UR, high UR was concentrated along the north-south axis, for instance in the governorates (2, 6, 14and 16). High CI was concentrated along the north-south axis, particularly in the northernareas and multiple governorates in southern areas, for instance, the governorates (2, 3, 5, 13, 14, 16 and 17). Based on *visual* inspection, the patterns formed by those governorates with highest ranking in UR and those with highest ranking in CI were somewhat identical. Second, many governorates were not observed visually as hot spots for both UR and CI, but after considering the information of their neighbours (i.e., calculating local Moran’s *I_i_* values), it can obviously see the patterns of hot spots, For example, governorates (3, 4, 5, and 12) for UR, and governorates (6 and 12) for CI. Third, based on global Moran index the clustering tendency showed that UR for each governorate can be spatially correlated with UR in neighbouring governorates, while the clustering tendency in CI was not found significant. Fourth, the significance of bivariate spatial correlation didn’t support the hypothesis of the association between the spatial patterns of CI and UR. Fifth, neighbouring governorates with high degree of inequality in UR seem to show higher levels in CI, for instance governorates (12 and 16). This was consistent with what Haining (2003) stated, the levels of such variable in area *i* was thought to be influenced by the levels of another variable not just in area *i* but also in its neighbouring areas. This supports the hypothesis that the degree of variations in UR between these governorates and their neighbours could somewhat influence CI.

The analytical approach used here accurately delineates governorates of high unemployment, and permits policy makers to develop strategies to minimize the difference between governorates. Policy which pays attention to area characteristics will reduce unemployment inequalities and consequently improve the health of the overall population. Additional research is needed to characterize more fully this relationship. Although unemployment and economic issues may seem beyond the usual bounds of health care, physicians and other health care professionals have the opportunity to recognize, treat and possibly prevent the adverse consequences of unemployment for their patients. Beyond caring for individuals, however, health care professionals can also pay an important role in collective action against unemployment by advocating better health.
